# Role of GLR-1 in Age-Dependent Short-Term Memory Decline

**DOI:** 10.1523/ENEURO.0420-23.2024

**Published:** 2024-03-29

**Authors:** Vaibhav Gharat, Fabian Peter, Dominique J.-F. de Quervain, Andreas Papassotiropoulos, Attila Stetak

**Affiliations:** ^1^Division of Molecular Neuroscience, Department of Biomedicine, University of Basel, Basel 4055, Switzerland; ^2^Research Cluster Molecular and Cognitive Neurosciences, University of Basel, Basel 4055, Switzerland; ^3^Division of Cognitive Neuroscience, Department of Biomedicine, University of Basel, Basel 4055, Switzerland; ^4^University Psychiatric Clinics, University of Basel, Basel 4002, Switzerland

## Abstract

As the global elderly population grows, age-related cognitive decline is becoming an increasingly significant healthcare issue, often leading to various neuropsychiatric disorders. Among the many molecular players involved in memory, AMPA-type glutamate receptors are known to regulate learning and memory, but how their dynamics change with age and affect memory decline is not well understood. Here, we examined the in vivo properties of the AMPA-type glutamate receptor GLR-1 in the AVA interneuron of the *Caenorhabditis elegans* nervous system during physiological aging. We found that both total and membrane-bound GLR-1 receptor levels decrease with age in wild-type worms, regardless of their location along the axon. Using fluorescence recovery after photobleaching, we also demonstrated that a reduction in GLR-1 abundance correlates with decreased local, synaptic GLR-1 receptor dynamics. Importantly, we found that reduced GLR-1 levels strongly correlate with the age-related decline in short-term associative memory. Genetic manipulation of GLR-1 stability, by either deleting *msi-1* or expressing a ubiquitination-defective GLR-1 (4KR) variant, prevented this age-related reduction in receptor abundance and improved the short-term memory performance in older animals, which reached performance levels similar to those of young animals. Overall, our data indicate that AMPA-type glutamate receptor abundance and dynamics are key factors in maintaining memory function and that changes in these parameters are linked to age-dependent short-term memory decline.

## Significance Statement

AMPA-type glutamate receptors are pivotal in synaptic transmission and plasticity, and their dysregulation is linked to neurological conditions and age-related cognitive decline. Despite their importance, the mechanisms regulating these receptors are not fully understood. In this study, we examined age-related changes in the AMPA-type glutamate receptor GLR-1 in *Caenorhabditis elegans* and assessed its impact on short-term memory decline. Our data show a significant reduction in both the abundance and local turnover of total and membrane-bound GLR-1 receptors as animals age. This decline closely correlates with a decreased performance in aversive olfactory memory tasks in older animals. Importantly, inhibiting GLR-1 degradation effectively mitigates short-term memory decline in aged animals.

## Introduction

Physiological aging prompts salient alterations in brain structure and function, which are presumed to underlie the observed decrease in various neuronal functions, with cognitive performance being notably impacted. Age-related changes in both cognitive and neurobiological domains are complex and vary between individuals, complicating the understanding of mechanisms underlying physiological and pathological cognitive decline. In today's society with a growing elderly population and a steep increase in neurodegenerative disorders such as Alzheimer's disease, it is on the other hand crucial to understand the impact of aging on brain structure and function to identify strategies to promote healthy aging and preserve cognitive functions in elderly people. An accumulating body of evidence suggests that age-related cognitive impairment is related to region-specific changes in neuronal morphology and synaptic plasticity (Henley and Wilkinson, 2013). More specifically, aging leads to decreased dendritic arborization and spine density, alterations in the composition and distribution of neurotransmitters, alterations in the structural and functional connections between different brain regions, and changes in the expression, trafficking, and function of neurotransmitter receptors such as glutamate receptors (Burke and Barnes, 2006; Bishop et al., 2010; Mattson and Arumugam, 2018).

In vertebrates, the glutamate receptor family includes three ionotropic (iGluR; NMDA, AMPA, and kainate) and metabotropic glutamate receptor I–III subfamilies (Traynelis et al., 2010). In the mammalian brain, the α-amino-3-hydroxy-5-methyl-4-isoxazolepropionic acid receptors (AMPARs) are the main receptors responsible for fast excitatory synaptic transmission (Bliss and Collingridge, 1993). AMPARs are composed of four subunits, GluA1–4, and can be organized into homo- or heterotetrameric complexes (Greger et al., 2017). Studies have shown that AMPAR trafficking and activity play a crucial role in various forms of synaptic plasticity and are essential for learning and memory formation in general (Huganir and Nicoll, 2013). Significant advances have been made over the last decades to broaden our understanding of the processes involved in regulating AMPA-type glutamate receptor dynamics at synapses during learning and memory; however, mechanisms that are affected during physiological aging remain largely unknown (Jurado, 2018).

The *Caenorhabditis elegans* (*C. elegans*) genome encodes at least 10 putative iGluR subunits which include members of the non-NMDA class (glr-1-8) and 2 NMDA class subunits (nmr-1, 2). GLR-1, one member of the iGluR subfamily, encodes a receptor subunit of a non-NMDA excitatory iGluR subtype and shows 40% homology with mammalian AMPA receptors GRIA2 and GRIA3 (Hart et al., 1995; Brockie et al., 2001a; Brockie and Maricq, 2003; Brockie, 2006). Of the many glutamate receptor types in nematodes, GLR-1 is the best investigated and has been linked to various behaviors such as locomotion, egg laying, and learning (Maricq et al., 1995; Morrison and Van Der Kooy, 2001; Rose et al., 2003). Previously, we have shown that GLR-1 is necessary for *C. elegans* associative learning and memory performance and demonstrated that cell-specific downregulation of GLR-1 activity in AVA interneuron selectively affects olfactory associative memory without impact on learning (Vukojevic et al., 2012). Furthermore, age-related memory decline in *C. elegans* is partially due to changes in AVA responsiveness (Fenyves et al., 2021). The command interneuron AVA plays a critical role in the nervous circuit regulating the reversal and backward movement in *C. elegans* and also seems to be a key regulator of short- and long-term memory (Brockie et al., 2001b; Stetak et al., 2009; Vukojevic et al., 2012; Hadziselimovic et al., 2014; Fenyves et al., 2021).

In the current study, we investigated the role of GLR-1 during incipient stages of age-dependent memory decline at the cellular level in the nematode *C. elegans*. To track GLR-1 abundance, distribution, transport, and turnover at synapses, we used a transgenic strain expressing functional GLR-1 protein fused to both superecliptic pHluorin (SEP, a pH-sensitive form of GFP) and mCherry at the extracellular N-terminal domain (SEP::mCherry::GLR-1) specifically in AVA interneuron (Hoerndli et al., 2013). This allowed us to simultaneously visualize global, as well as plasma membrane–inserted receptor levels, which is of particular importance as AMPARs have been described as having a highly dynamic turnover at the synapses (Choquet and Triller, 2013). Our results show that GLR-1 abundance declines significantly with age. Moreover, aging influences GLR-1 dynamics at the synapses by affecting the local short–distance synaptic delivery and insertion of GLR-1 into the cell membrane. The observed effect on GLR-1 abundance highly correlates with a decline in memory performance during aging. Finally, we demonstrate that reverting the decline of GLR-1 abundance in aged animals restores GLR-1 dynamics and ultimately improves memory performance.

## Materials and Methods

### General methods and strains used

Standard methods were used for maintaining and manipulating *C. elegans* (Brenner, 1974). *C. elegans* Bristol strain, variety N2, was used as the wild-type reference strain. The experiments were performed with synchronized worm populations of young adult hermaphrodites. Gravid adult worms were washed with M9 and bleached using an alkaline hypochlorite solution (0.5 M NaOH, 5% HOCl). Eggs were allowed to hatch on nonseeded CTX agar plates overnight at 20°C. The synchronized L1 worms were then transferred and always grown on NGM-OP50 plates to adulthood at 20°C. The synchronized L1 worms were grown for ∼70 h at 20°C, until they reached adulthood and started reproducing, and referred to as 1-d-old/young adult worms. Subsequently, for every additional 24 h of growth at 20°C, we counted one more day in their age. For all the aging experiments, worms were transferred away from progeny to fresh plates every day. All the experiments were conducted at RT in acclimatized rooms. The imaging and the behavioral data were collected in a nonblinded manner.

The *C. elegans* alleles and strains used in this study were as follows:

*msi-1(os1), akIs201; Prig-3::SEP GFP::mCherry::glr-1, utrIs51[Prig-3::SEP GFP::mCherry::glr-1(4KR)], msi-1(os1); akIs201[Prig-3::SEP GFP::mCherry::glr-1], akIs141; Prig-3::HA::glr-1::gfp*.

### Olfactory memory assays

Short-term learning and memory were assessed as described previously with some modifications (Stetak et al., 2009). Briefly, well-fed young adult worms were exposed for 1 h to starvation in the presence of 2 μl undiluted chemoattractant diacetyl (DA) spotted on the lid of 10 cm CTX plates (5 mM KH_2_PO_4_/K_2_HPO_4_, pH 6.0, 1 mM CaCl_2_, 1 mM MgSO_4_, 2% agar), and their attraction to DA was tested prior (naive), directly after (conditioned), and following a 1 h rest in the absence of DA and food (1 h delay). Naive and conditioned worms were given a choice between a spot of 0.1% DA in ethanol with 20 mM sodium azide and a counter spot with ethanol and sodium azide. After a delay time, the animals were counted, and the chemotaxis index was calculated as described previously (Bargmann et al., 1993). A total of 50–200 animals were used in each technical and biological replicate. In the case of memory assays with aged animals, learning index [(CI_Naive_ − CI_conditioning_)/CI_Naive_] and memory index [(CI_Naive_ − CI_1h delay_)/CI_Naive_] were used to normalize the age-dependent chemotaxis decline.

### Nose touch assays

The assay was conducted as previously described (Hart et al., 1999). The nose touch assay was carried out on a thin layer of bacteria (OP50 culture). Worms were transferred onto the plates and allowed to recover for approximately 30 min. An eyelash hair, glued to a glass probe, was placed in front of a forward-moving worm. Responses were recorded as reversals if the worm immediately initiated a backward movement. No response was recorded if the worm continued moving forward and crawling under, above, or along the eyelash. Each individual animal was tested five times with ∼30 s intervals. A minimum of 10 worms per strain were tested on Day 1, Day 2, and Day 3. The average response of each worm to the five touches was then used to determine the average response of each strain for that specific day.

### Fluorescence microscopy

Worms were mounted on 7% agarose pads with 3 µl of polystyrene beads (Polybead, catalog #00876-15, Polysciences), unless otherwise indicated. Images were acquired using Leica point scanning confocal SP8 with 63×/1.4 oil Plan Apo objective using 488 and 552 nm excitation lasers. All the microscopy data were recorded in a proximal (posterior to AVA chiasma) and in the most distal region of AVA projections, along the *z*-axis, unless otherwise indicated.

### GLR-1 abundance analysis

The *akIs201[Prig-3::SEP::GFP::mCherry::glr-1]*, *utrIs51[Prig-3::SEP::GFP::mCherry::glr-1(4KR)]* and *msi-1(os1); akIs201[Prig-3::SEP::GFP::mCherry::glr-1]* strains were used to conduct intensity analysis on adult animals at Day 1 and Day 3. All recorded images were processed and quantified using FIJI. Total fluorescence signal intensities in a ∼40-µm-long region (proximal and distal) of AVA projections were quantified by drawing the region of interest (ROI) on the maximal projection images of confocal stacks. The background fluorescence (i.e., outside of the AVA) from each maximum projection was then subtracted from the average fluorescence of the ROI. The measured raw intensities were normalized to the mean of 1-d-old adult signal from the respective strain.

### Fluorescence recovery after photobleaching (FRAP)

The *akIs201[Prig-3::SEP::GFP::mCherry::glr-1], utrIs51[Prig-3::SEP::GFP::mCherry::glr-1(4KR)]* and *msi-1(os1); akIs201[Prig-3::SEP::GFP::mCherry::glr-1]* strains were used to conduct FRAP on adult animals at Day 1 and Day 3. Animals were immobilized with 3 μl of a mixture containing equal measures of polystyrene beads (Polybead, catalog #00876-15, Polysciences) and 30 mM muscimol (catalog #195336, MP Biomedicals) on 7% agarose pads. A prebleached image stack was acquired using the 488 and 552 nm laser. Bleaching of the ROI was done with 488 and 552 nm laser using four iterations (∼1 s total illumination) at 100% laser energy. The imaging region was photobleached, and immediately after, one image stack (with 488 and 552 nm excitation) was acquired for the 0 min timepoint. This was repeated at 2, 4, 6, and 8 min following the photobleaching of the imaging region. The recorded images were processed using FIJI to generate maximal projection. The signal intensities were manually quantified by drawing ROIs around the bleached spot. To compensate for the photobleaching over the time period, the recovery fluorescence signal at the bleached spot was normalized with the signal from a nonbleached area within the image. The same regions were used to quantify the membrane-bound (SEP::GFP) and total GLR-1 (mCherry) FRAP signals.

### Transport kymographs

Mutants from *akIs141[Prig-3::HA::glr-1::gfp]* strains were mounted for imaging as described above. Once the neurons were located using the 64× objective and a 488 nm excitation laser (solid-state laser, 20 mW) at 2% power, a proximal section of the neurites was photobleached using 200 iterations (∼6 s total illumination) at 100% laser energy. Transport images were acquired by taking a streaming movie in projected Z section of 0.44 µm (number of steps, 2). Immediately following photobleaching, a 600-frame image stream was collected with the 488 nm excitation laser at 8% power at a frame rate of 10 fps.

Kymographs were generated using the Multi Kymograph tool in FIJI with a 29-pixel line width, and the kymographs were analyzed using KymoButler to yield the number of transport events and velocity (Jakobs et al., 2019).

### Real-time reverse transcription–quantitative PCR

RNA expression levels were measured from synchronized 1- and 3-d-old worms. For every sample, 10 animals were collected in 2 μl worm lysis buffer (50 mM KCl, 10 mM Tris–HCl, pH 8.2, 2.5 mM MgCl_2_, 0.45% NP-40, 0.45% Tween 20, 0.01% gelatin) supplemented with 60 μg/ml proteinase K and digested for 1 h at 50°C followed by 10 min at 95°C. Immediately after digestion, the samples were processed with Maxima First Strand cDNA Synthesis Kit (Thermo Fisher Scientific) according to the manufacturer's recommendation. Real-time quantitative PCR (qPCR) was performed with gene-specific primers (Extended Data Table 1) using SYBR FAST Kit (Kapa Biosystems) in a Rotor-Gene 6000 instrument (Corbett Research). Real-time qPCR was performed with gene-specific primers (Extended Data Table 1) using SYBR FAST Kit (Kapa Biosystems) according to the manufacturer's recommendations in a Rotor-Gene 6000 instrument (Corbett Research). The total RNA levels were normalized to the expression levels of *tba-1* and *cdc-42*. Fold differences were calculated using the ΔΔC_t_ method.

10.1523/ENEURO.0420-23.2024.t1 Extended Data Table 1 Summary table of the primers used for quantitative real-time PCR. Download Extended Data Table 1, XLS file.

### Statistical analysis

All data and statistical analyses were carried out using Prism 9. Data are represented as Tukey's boxplot unless otherwise indicated. Outlier analysis was carried out using the ROUT method with a false discovery rate set to 1%. Detected outliers were excluded from the data analysis. Normality of the data was tested using D’Agostino and Pearson’s normality test. Main effects and interaction terms were tested using ANOVA. Statistical tests for significance were done with *F* tests using sum-of-square type I. The *p* value threshold was set to nominal significance (*p* < 0.05). Pairwise group comparison was tested using post hoc *t* tests corrected for multiple comparisons using Tukey’s correction (*p* < 0.05). All figures were created using Adobe Illustrator. For detailed statistics, see Extended Data Table 2.

## Results

### Physiological aging decreases GLR-1 abundance in AVA neuron

**Figure 1.**
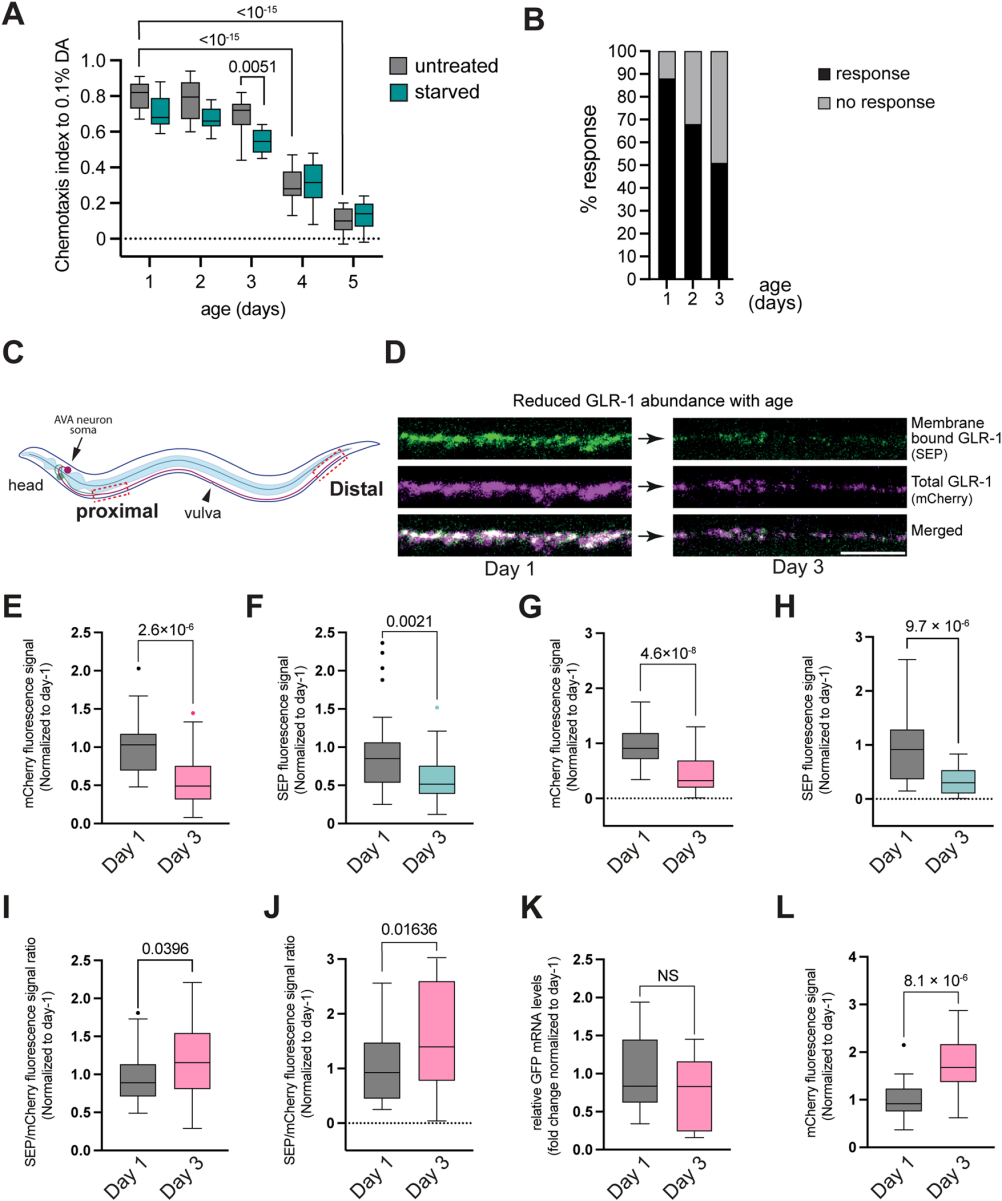
GLR-1 abundance declines in wild-type worms during aging. ***A***, Age-dependent chemotaxis decline toward DA in wild-type worms. ***B***, Nose touch response of young, 2-d-old, and 3-d-old wild-type adult worms. ***C***, Scheme illustrating the location for in vivo imaging of GLR-1 protein intensities and dynamics in the AVA neuron. ***D***, The GLR-1 receptor abundance was measured using confocal microscopy in transgenic worms in the wild-type background. Representative image of the membrane-bound and total GLR-1 content of the AVA axon in young (left) and 3-d-old (right) wild-type animals. ***E–H***, Box plots displaying quantification of the fluorescence intensity of (***E***) total (mCherry) and (***F***) membrane-bound (SEP) GLR-1 receptors normalized to Day 1 signal in the proximal part of the axon of the AVA neuron; (***G***) total (mCherry) and (***H***) membrane-bound (SEP) GLR-1 level in the distal parts of AVA axon of SEP::mCherry::GLR-1(wt) worms. ***I***, ***J***, Membrane-bound versus total GLR-1 ratio in the proximal (***I***) and distal part (***J***) of AVA axon in 1-d-old (left) and 3-d-old (right) wild-type animals; (***K***) *glr-1* RNA levels were measured with real-time qRT-PCR in 1- and 3-d-old SEP::mCherry::GLR-1(WT) strain. Expression levels were normalized to *tba-1* and *cdc-42*. ***L***, Fluorescence intensity levels of the mCherry (total GLR-1) signal in young and aged SEP::mCherry::GLR-1(WT) animals in the cell body of the AVA neuron. Scale bars, 5 µm. Data on (***A***) and (***E–L***) are visualized with Tukey's boxplots. See Extended Data Table 2 for detailed statistical information.

Physiological age-dependent memory decline is readily observable in *C. elegans*, with 2-d-old worms already displaying a decline in olfactory associative memory (Kauffman et al., 2010; Fenyves et al., 2021; Mastrandreas et al., 2022). Although the motility remains intact for a longer time period (Kauffman et al., 2010) and the pharyngeal pumping rate doesn't decline until Day 4 (Huang et al., 2004; Chow et al., 2006), the chemotaxis toward DA significantly declines after Day 3 (Fig. 1*A*) and is lost at timepoints previously used in neurodegenerative studies (Wirak et al., 2022). Finally, the rate of the age-related chemotaxis decline toward DA was not strongly altered by starvation (Fig. 1*A*).

10.1523/ENEURO.0420-23.2024.t2 Extended Data Table 2 Detailed description of the statistical data for figures 1-8. Download Extended Data Table 2, XLS file.

Previously, *glr-1* mutants have also been shown to exhibit defects in response to touch of the nose (Hart et al., 1995; Maricq et al., 1995). To investigate whether the age-related reduction in GLR-1 is a general phenomenon affecting different GLR-1 dependent on behaviors including nose touch response, as well as learning and memory, we first examined the nose touch response in wild-type animals on Days 1, 2, and 3. We observed a gradual reduction in the nose touch response with age, correlating with the observed decline in GLR-1 abundance suggesting a general age-dependent reduction of the GLR-1 receptor levels in *C. elegans* nervous system (Fig. 1*B*).

Next, to capture the molecular changes that may correlate with the previously observed memory decline, we chose to compare GLR-1 properties between 1- and 3-d-old animals allowing us to simultaneously study early changes of GLR-1 dynamics during aging and their effect on the physiological cognitive decline. In order to establish a link between physiological age-dependent cognitive decline and glutamate signaling, we first investigated whether GLR-1 receptor levels in the AVA interneuron decrease during Day 1 and Day 3 of adulthood, which may contribute to memory decline. To measure possible GLR-1 abundance changes with age, first we quantified the total and membrane-bound GLR-1 levels in the SEP::mCherry::GLR-1 strain with confocal microscopy.

AVA interneurons project their axon along the entire length of the ventral nerve chord and receive considerable synaptic inputs from many different neurons in the anterior and posterior parts (White et al., 1986). Unfortunately, the circuitry involved in associative memory formation in *C. elegans* is still not fully elucidated, and it remains unknown which of the synaptic inputs are the most relevant. Therefore, we decided to measure the signal intensity of SEP and mCherry both in the proximal and also at the distal region relative to the soma of the AVA neuron (Fig. 1*C*). These two regions likely represent different synaptic inputs (such as PVC input in the distal region of the AVA axon) that allowed us to investigate possible differences in GLR-1 abundance along the axon in 1- and 3-d-old adult animals (Fig. 1*D*). In this experimental setup, we found a significant reduction in the total GLR-1 amount in 3-d-old animals in AVA interneurons (Fig. 1*E*). Additionally, we also observed a significant drop of the membrane-bound GLR-1 levels (Fig. 1*F*), represented by the SEP signal. This was true for both the proximal and distal part of the AVA axon suggesting a global rather than localized subcellular decrease in GLR-1 levels (Fig. 1*G*,*H*).

We further analyzed whether there are any changes in the ratio of membrane-bound versus total GLR-1 and found that the ratio significantly increased with age in both the proximal and the distal part (Fig. 1*I*,*J*). Thus, we observed a decrease in both membrane-bound and total GLR-1 receptor; however, the decrease in membrane-bound receptor is less pronounced.

To understand the cause of the observed decrease in GLR-1 abundance, we tested if aging alters mRNA levels or the translation rate of GLR-1. To quantify age-related AVA interneuron-specific changes in *glr-1* mRNA expression, we took advantage of the SEP::GFP sequence in SEP::mCherry::GLR-1 strain and measured the mRNA levels coding the fusion protein with quantitative RT-PCR. This experiment showed no significant decline in *glr-1* mRNA levels with age (Fig. 1*K*).

In addition, we also measured the amount of GLR-1 protein in the soma of the AVA neuron in young and aged animals by measuring the intensity of the mCherry signal in SEP::mCherry::GLR-1 strain, which corresponds to the total GLR-1 amount. Interestingly, we found that GLR-1 level is not reduced but, in contrast, significantly increased in the AVA soma with aging (Fig. 1*L*). This suggests that the observed overall decrease in GLR-1 abundance is not caused by a reduction of GLR-1 protein synthesis in the cell body.

**Figure 2.**
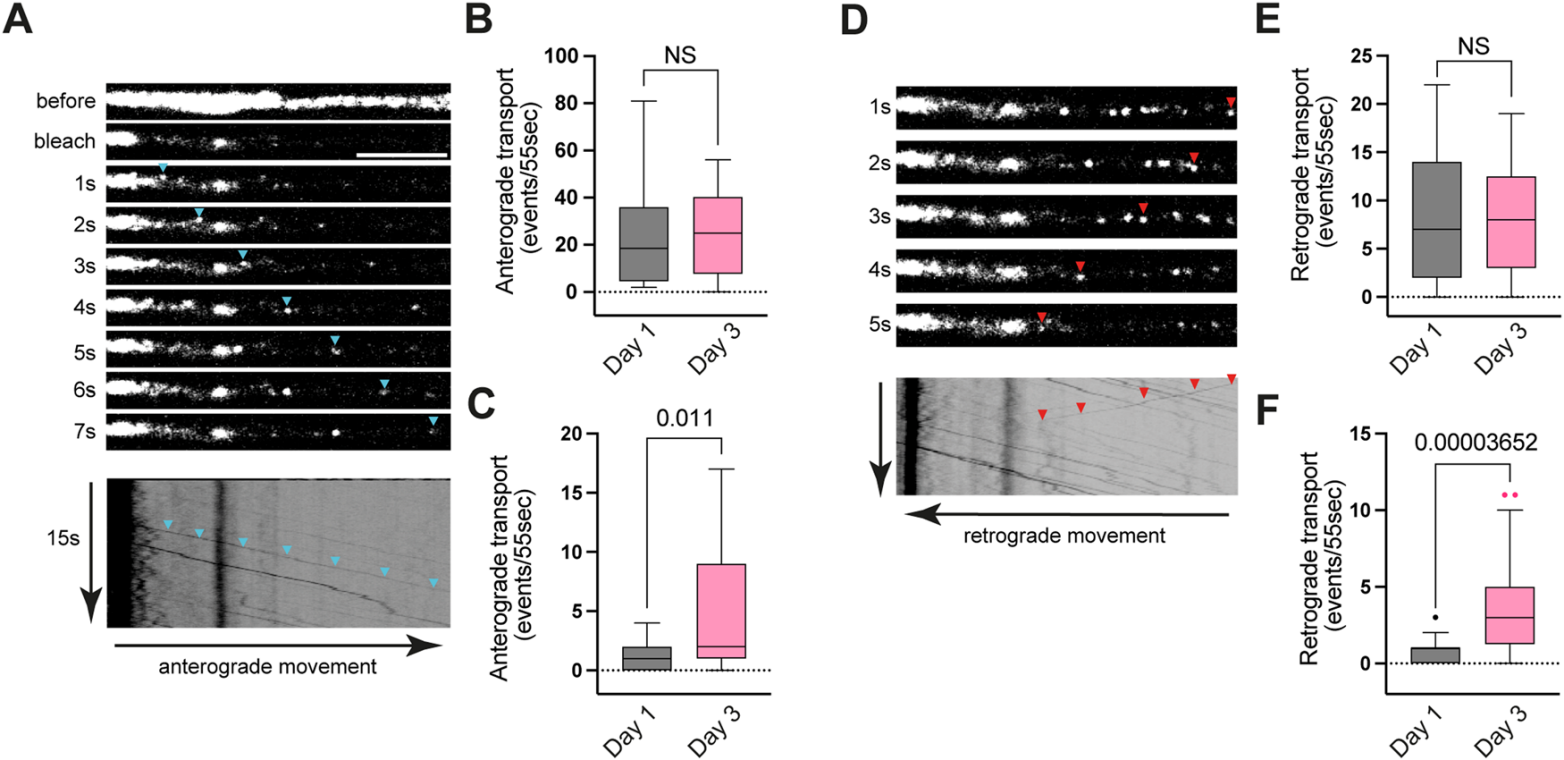
GLR-1 transport does not decline in wild-type worms at Day 3. ***A***, ***D***, Time-lapse images of GLR-1 vesicle movements along the axon of the AVA neuron. The moving vesicle is highlighted with arrowheads. The bottom panel shows a kymogram of all merged timeframes where transport events can be seen as oblique lines crossing. ***A***, Anterograde transport from the soma (vesicle; blue arrowhead). ***D***, Representative example of the retrograde transport (vesicle; red arrowhead). ***B***, Average number of anterograde and (***C***) retrograde transport events in the proximal part of AVA. ***E***, Average number of anterograde and (***F***) retrograde transport events in the distal part of AVA of SEP::mCherry::GLR-1(wt) animals. Scale bars, 5 µm. Data on ***B***, ***C***, ***E***, and ***F*** are visualized with Tukey's boxplots. See Extended Data Table 2 for detailed statistical information.

Next, we wondered whether the observed decline in GLR-1 abundance is due to a reduction in long-distance transport of GLR-1 from the soma to the synaptic sites instead of a decrease in protein synthesis. By using real-time streaming confocal microscopy, we investigated anterograde and retrograde GLR-1 transport frequency to and from the AVA cell body and whether it changes with aging (Fig. 2*A*,*D*). Interestingly, we did not observe any significant decline in the frequency of anterograde and retrograde transport in the proximal part, and on the contrary we observed an increase in the anterograde and retrograde transport frequency in the distal part during aging (Fig. 2*B,C,E,F*).

Taken together, our results suggest that GLR-1 synthesis and long-distance transport along the axon are not affected by age during the early physiological cognitive decline and are likely not the key driving mechanism of the age-dependent reduction of GLR-1 at the synapses. Thus, the observed reduction of receptor content in AVA is more likely due to age-related differences in local receptor dynamics.

### Dynamics of glr-1 change with age

**Figure 3.**
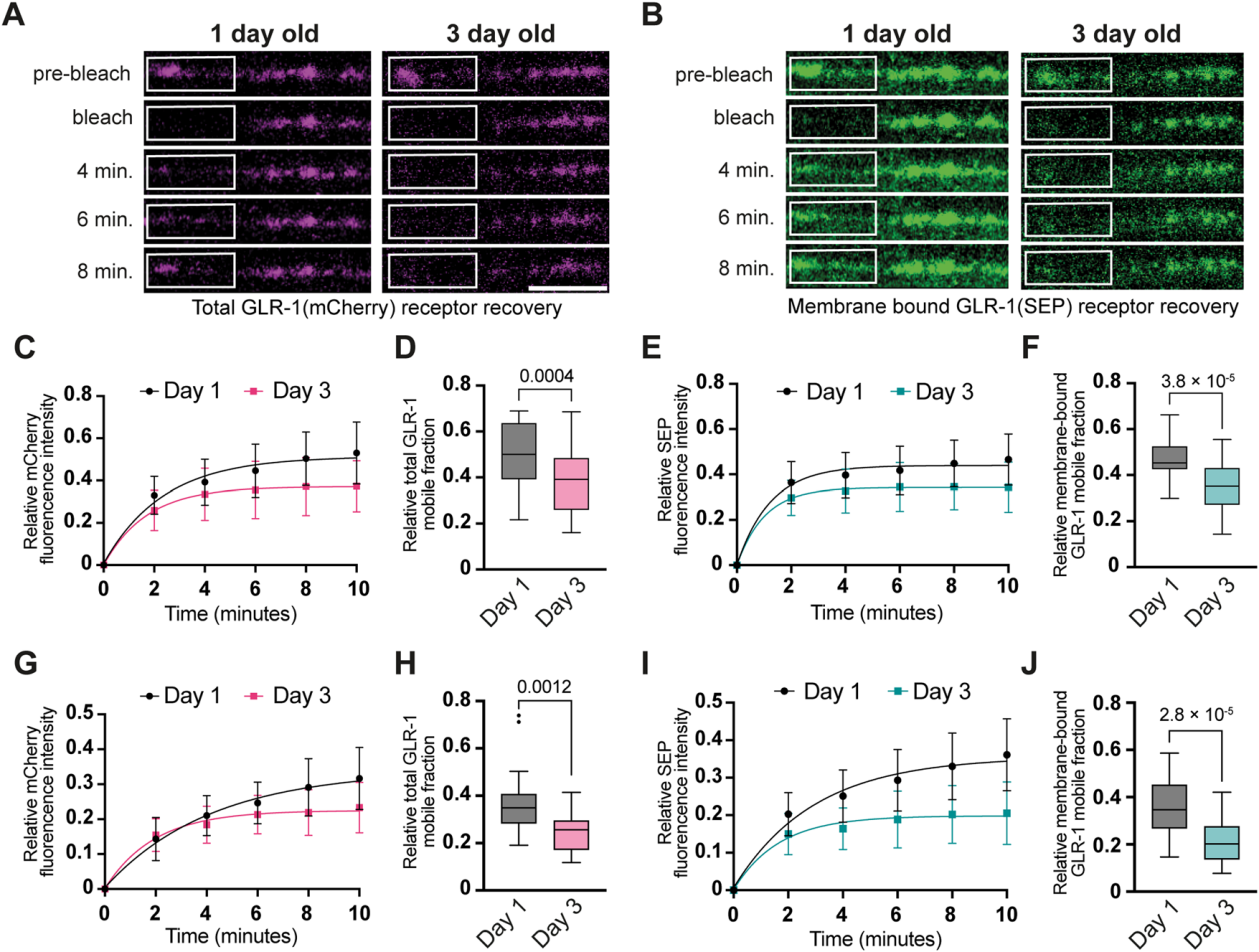
Age-dependent GLR-1 dynamics decline in wild-type worms. ***A***, ***B***, Representative image of the FRAP recovery of the membrane-bound (***A***) and total GLR-1 (***B***) content of the AVA axon in young and aged wild-type animals over time. ***C***, ***E***, Percentage FRAP recovery in the proximal part for (***C***) mCherry fluorescence over time at Day 1 (plateau, 0.5136) and at Day 3 (plateau, 0.3718) and (***E***) SEP fluorescence at Day 1 (plateau, 0.4395) and at Day 3 (plateau, 0.3428; *n* > 22 for each group). ***D***, ***F***, Quantity of the mobile fraction of (***D***) total GLR-1 and (***F***) membrane-bound GLR-1 during aging in the proximal part of the AVA neuron. ***G***, ***I***, Percent recovery of (***G***) mCherry fluorescence after photobleaching over time at Day 1 (plateau, 0.34) and at Day 3 (plateau, 0.2250) and (***I***) SEP fluorescence at Day 1 (plateau, 0.3555) and at Day 3 (plateau, 0.1983; *n* > 20 for each group) in the distal part. ***H***, ***J***, Relative quantity of the mobile fraction of (***H***) total GLR-1 and (***J***) membrane-bound GLR-1 during aging in the distal part of the AVA neuron during aging. Scale bar, 5 µm. Data on ***D***, ***F***, ***H***, and ***J*** are visualized with Tukey's boxplots, and ***C***, ***E***, ***G***, and ***I*** show mean ± SEM. See Extended Data Table 2 for detailed statistical information.

To gain insight into the local receptor dynamics in young and aged animals, we performed FRAP experiments along the AVA axons using the SEP::mCherry::GLR-1 strain. We monitored the recovery of mCherry (total GLR-1; Fig. 3*A*) and SEP (membrane bound; Fig. 3*B*) after photobleaching to quantify the local short-distance delivery of new GLR-1 to synaptic sites and GLR-1 membrane integration, respectively, both in the proximal and distal region of the AVA axon. After photobleaching, the mCherry signal is bleached, while SEP fluorescence of nonmembrane-bound GLR-1 is protected from photobleaching as the receptor is in a quenched state inside the acidic endosomes (Kennedy et al., 2010; Hoerndli et al., 2015). Quantification of the GLR-1::mCherry and the GLR-1::SEP FRAP signal demonstrated that at the proximal part of AVA, both the amount of the total GLR-1 and the membrane-bound GLR-1 mobile fraction reduced significantly with age (Fig. 3*C*–*F*). A similar effect was observed when analyzing the GLR-1::mCherry and the GLR-1::SEP FRAP for the distal part of AVA (Fig. 3*G*–*J*). These experiments suggest that during aging the GLR-1 synaptic receptor mobility and local turnover are significantly decreased compared with young animals.

### GLR-1 abundance is maintained with age in ubiquitination-defective mutants

**Figure 4.**
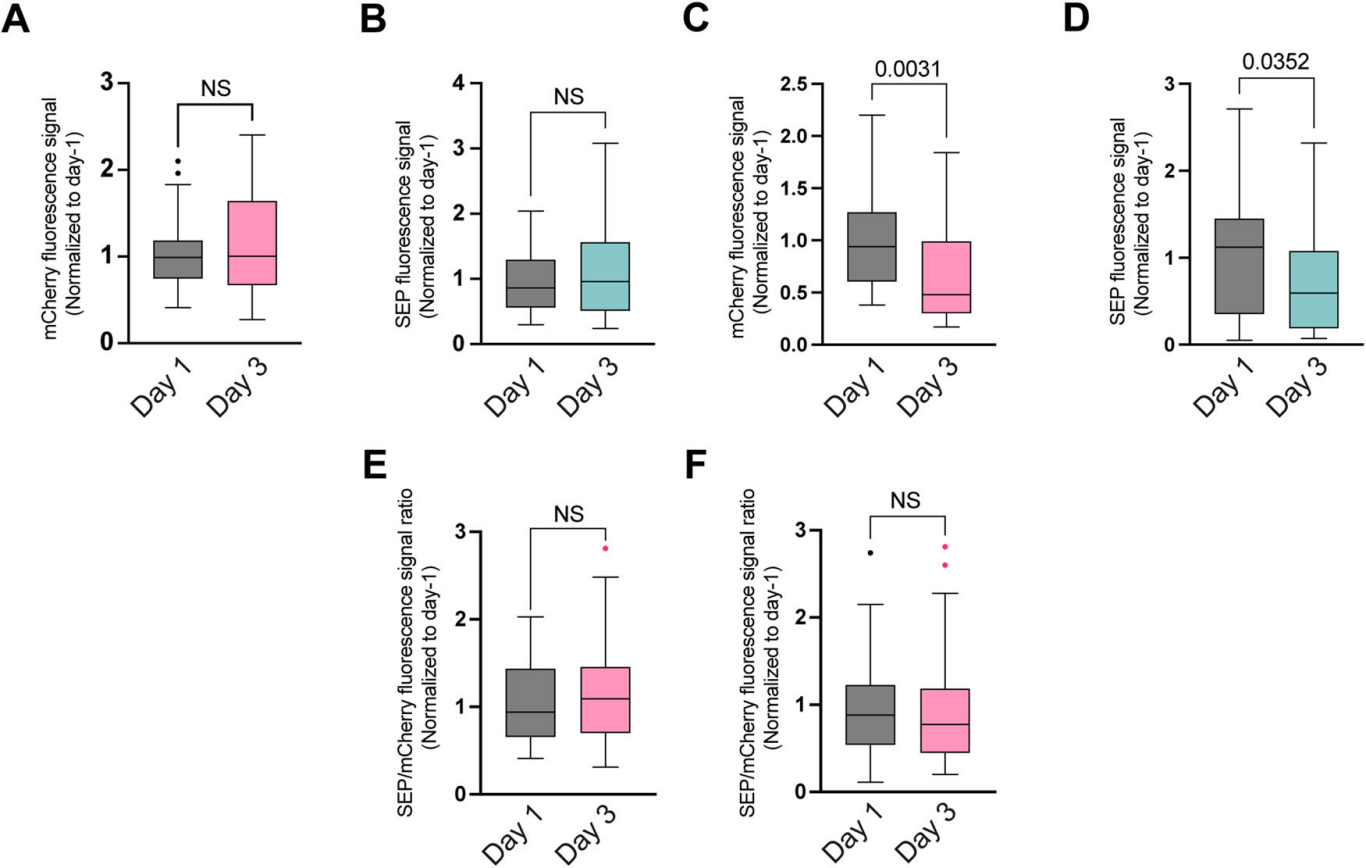
GLR-1 abundance is maintained with age in ubiquitination-defective mutants. ***A***, ***B***, Box plots displaying quantification of the fluorescence intensity of (***A***) total GLR-1 and (***B***) membrane-bound receptors normalized to Day 1 signal in the proximal part of the axon of AVA neuron of 4KR mutant worms. ***C***,***D***, Receptor levels of (***C***) total GLR-1 and (***D***) membrane bound in the distal part of AVA axon of 4KR mutant worms. ***E***,***F***, Membrane-bound versus total GLR-1 ratio in the proximal (***E***) and distal part (***F***) of the AVA axon in 1-d-old (left) and 3-d-old (right) animals carrying a ubiquitination-defective GLR-1 reporter. Data on ***A–F*** are visualized with Tukey's boxplots. See Extended Data Table 2 for detailed statistical information.

To further test our hypothesis that the reduced GLR-1 synaptic abundance is related to changes in local GLR-1 dynamics, we used a transgenic strain expressing SEP::mCherry::GLR-1(4KR), a ubiquitination-defective variant of GLR-1 that is predicted to increase the number of cell-surface synaptic receptors (Burbea et al., 2002; Grunwald et al., 2004). First, we measured GLR-1 abundance in 4KR mutants, and the ubiquitination-defective GLR-1 mutant showed no age-dependent decrease in the abundance of total GLR-1 in the proximal part (Fig. 4*A*) of AVA neurons and a marginal, albeit significant, reduction in the more distal part (Fig. 4*C*). The same trend was observed for the abundance of membrane-bound GLR-1 (Fig. 4*B*,*D*).

We also calculated the changes in the ratio of membrane-bound versus total GLR-1 in SEP::mCherry::GLR-1(4KR) strain. Contrary to the observations in the SEP::mCherry::GLR-1(WT) strain, we found no significant differences in the ratio with age neither in the proximal nor in the distal part of the AVA axon (Fig. 4*E*,*F*).

**Figure 5.**
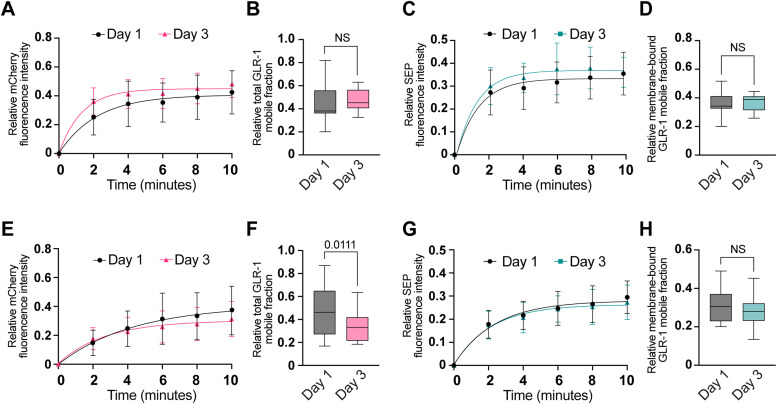
GLR-1 dynamics is maintained with age in ubiquitination-defective mutants. ***A***, ***C***, Percent fluorescence recovery over time after photobleaching of (***A***) mCherry at Day 1 (plateau, 0.4062) and at Day 3 (plateau, 0.4493) and (***C***) SEP::GFP at Day 1 (plateau, 0.3358) and at Day 3 (plateau, 0.3728; *n* > 13 for each group) in the proximal part of AVA neuron. ***B***, ***D***, Mobile fraction of (***B***) total GLR-1 and (***D***) membrane-bound GLR-1 during aging in the proximal part of the AVA neuron. ***E***, ***G***, Percent fluorescence recovery of (***E***) mCherry after photobleaching over time at Day 1 (plateau, 0.4079) and at Day 3 (plateau, 0.3024) and (***G***) SEP at Day 1 (plateau, 0.2801) and at Day 3 (plateau, 0.2632; *n* ≥ 20 for each group) in the distal part. ***F***, ***H***, Quantification of the mobile fraction of (***F***) total GLR-1 and (***H***) membrane-bound GLR-1 in the distal part of the AVA neuron during aging. Data on ***B***, ***D***, ***F***, and ***H*** are visualized with Tukey's boxplots. Panels ***A***, ***C***, ***E***, and ***G*** show mean ± SEM. See Extended Data Table 2 for detailed statistical information.

Secondly, we measured GLR-1 dynamics using FRAP in 4KR mutants, and, consistent with our previous results, we did not observe altered GLR-1 dynamics in ubiquitination-defective mutants with age, except for a subtle decrease of recovery dynamics after photobleaching of the total GLR-1 in the distal part of the AVA axon (Fig. 5*A*–*H*). However, this could be attributed to the observed decrease in the abundance of total GLR-1 signal in the distal part of the AVA axon, which may result from reduced local receptor turnover, lateral receptor diffusion, and availability with age (Fig. 4*C*). Altogether, our data suggest that when degradation of GLR-1 is inhibited and receptor abundance does not markedly decrease with age, receptor dynamics also remain relatively intact. However, our experimental setup cannot rule out that besides degradation, other important mechanisms like lateral receptor diffusion and local receptor synthesis could also be affected during aging.

### GLR-1 abundance, dynamics, and memory performance remain unchanged during aging in *msi-1(lf)* mutants

**Figure 6.**
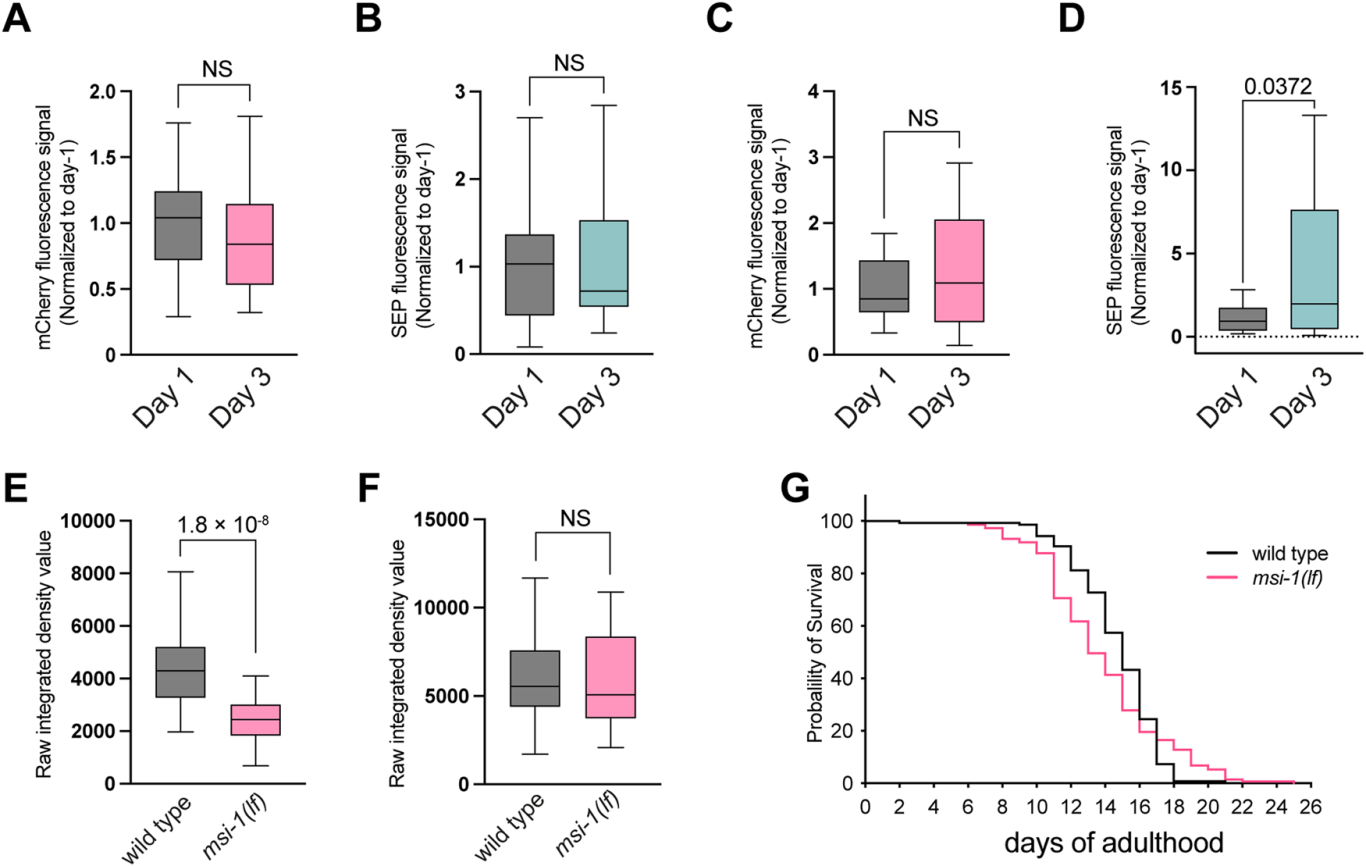
GLR-1 abundance remains unchanged in *msi-1(lf)* mutants with aging. ***A***, ***B***, Quantification of the fluorescence intensity of (***A***) total and (***B***) membrane-bound GLR-1 receptors normalized to Day 1 signal in the proximal part of the axon of AVA neuron of *msi-1(lf)* mutant worms. ***C***, ***D***, Receptor levels of (***C***) total GLR-1 and (***D***) membrane bound in the distal part of AVA axon. ***E***, ***F***, mCherry fluorescence intensity was compared at Day 1 between SEP::mCherry::GLR-1(WT) and *msi-1(lf)* mutant animals in the (***E***) proximal and (***F***) distal part of the AVA axon. ***G***, Life span of wild-type and *msi-1(lf)* mutant animals. Data on ***A*–*F*** are visualized with Tukey's boxplots. See Extended Data Table 2 for detailed statistical information.

In a previous study, we identified the RNA-binding protein musashi (MSI-1) which promotes forgetting in *C. elegans* and has been shown to regulate the removal of AMPA receptors from synapses during long-term depression (LTD; Rocca et al., 2008; Hadziselimovic et al., 2014). Interestingly, *msi-1(lf)* mutants have improved short- and long-term memory retention when tested at Day 1 of adulthood and also do not show long-term age-dependent memory decline when 2-d-old animals are trained and tested for 24 h recovery on Day 3 (Mastrandreas et al., 2022). Therefore, we compared GLR-1 abundance and dynamics in the *msi-1* loss-of-function mutants and compared with the wild-type animals. As expected, we did not observe any age-dependent decrease in total (Fig. 6*A*) or in membrane-bound GLR-1 (Fig. 6*B*) levels in the proximal part in *msi-1(lf)* mutants. Similarly, we observed sustained levels of total (Fig. 6*C*) as well as membrane-bound GLR-1 (Fig. 6*D*) receptors between 1-d-old and 3-d-old mutants in the distal part of the AVA axon. To understand if *msi-1(lf)* mutation has any effect on the baseline GLR-1 abundance, we compared the mCherry fluorescence intensity values between 1-d-old SEP::mCherry::GLR-1(WT) and *msi-1(lf)* mutant animals. To our surprise, we observed significantly reduced baseline total GLR-1 abundance in *msi-1(lf)* mutants in the proximal part (Fig. 6*E*). However, the baseline levels stayed unchanged in the distal part of the AVA axon (Fig. 6*F*).

**Figure 7.**
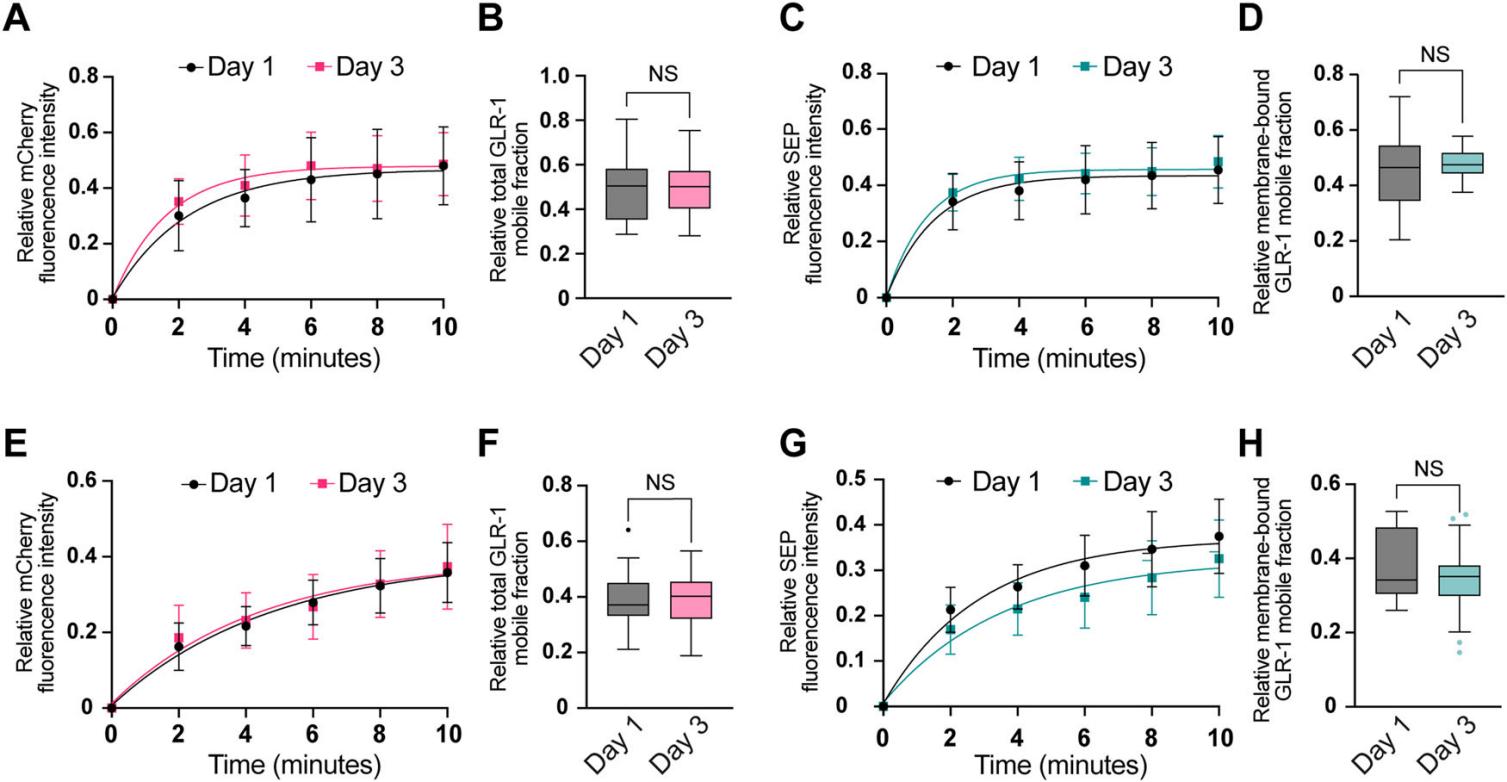
GLR-1 dynamics remain unchanged in *msi-1(lf)* mutants with aging. ***A***, ***C***, Fluorescence recovery following photobleaching of (***A***) mCherry at Day 1 (plateau, 0.4681) and at Day 3 (plateau, 0.4793) and (***C***) SEP at Day 1 (plateau, 0.4341) and at Day 3 (plateau, 0.4562; *n* > 16 for each group) in the proximal part of AVA neuron. ***B***, ***D***, Box plots displaying mobile fraction of (***B***) total GLR-1 and (***D***) membrane-bound GLR-1 during aging in the proximal part of the AVA neuron. ***E***, ***G***, Percent fluorescence recovery of (***E***) mCherry after photobleaching over time at Day 1 (plateau, 0.3988) and at Day 3 (plateau, 0.3932) and (***G***) SEP::GFP at Day 1 (plateau, 0.37) and at Day 3 (plateau, 0.3240; *n* > 17 for each group) in the distal part. ***F***, ***H***, Relative quantity of the mobile fraction of (***F***) total GLR-1 and (***H***) membrane-bound GLR-1 during aging in the distal part of the AVA neuron of *msi-1(lf)* mutants at Day 1 and Day 3. Data on ***B***, ***D***, ***F***, and ***H*** are visualized with Tukey's boxplots. Panels ***A***, ***C***, ***E***, and ***G*** show mean ± SEM. See Extended Data Table 2 for detailed statistical information.

Furthermore, FRAP analysis for mCherry and SEP signal revealed that GLR-1 dynamics for total and membrane-bound GLR-1 do not change with age in *msi-1 (lf)* mutants (Fig. 7*A*–*H*).

**Figure 8.**
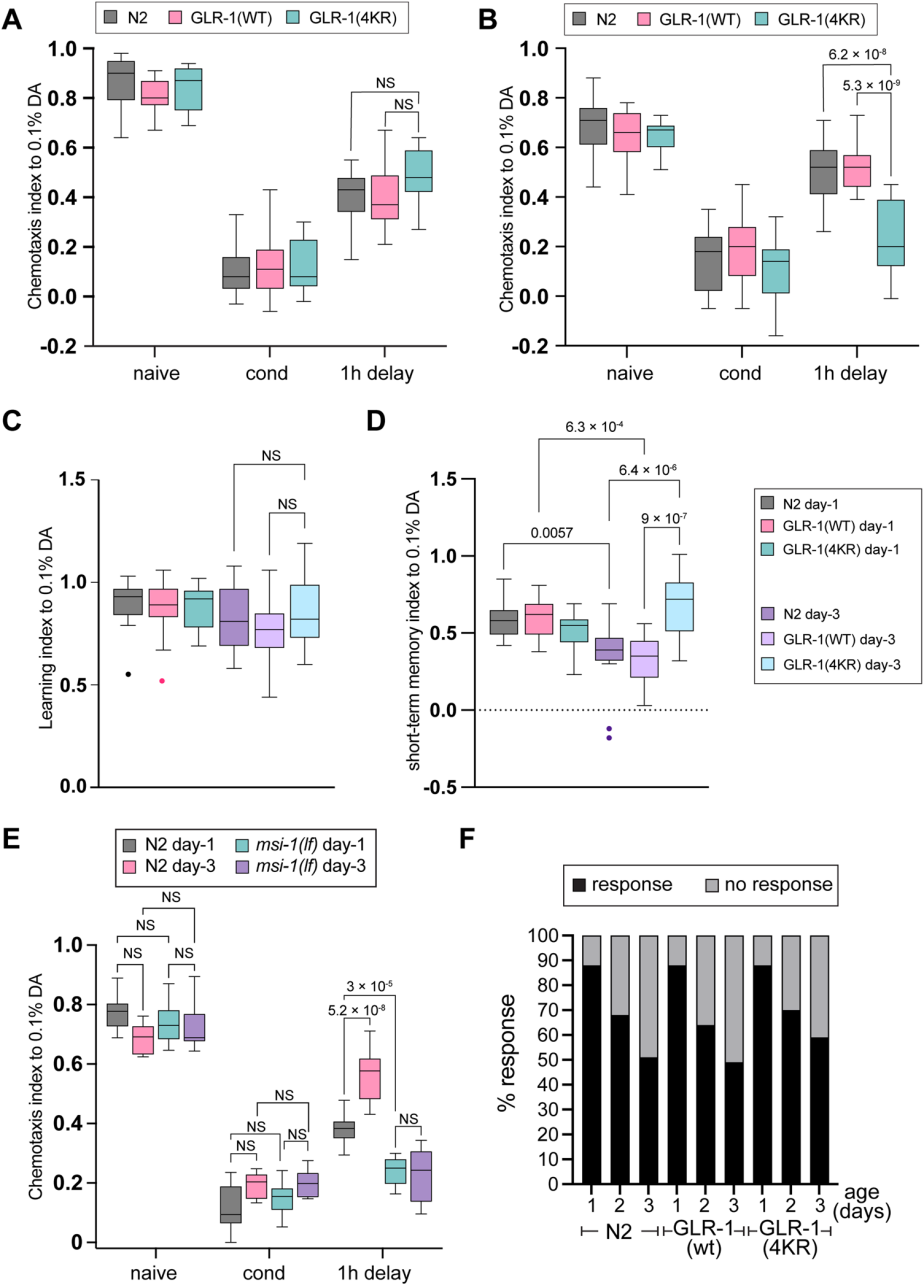
Memory is maintained in GLR-1(4KR) mutants with age. ***A***, ***B***, Short-term aversive olfactory memory assay of wild-type, transgenic animals carrying either SEP::mCherry::GLR-1(wt) or SEP::mCherry::GLR-1(4KR) using DA was tested in (***A***) young adult and (***B***) 3-d-old worms. ***C***, Learning index [(CI_Naive_ − CI_conditioning_) / CI_Naive_] or (***D***) memory index [(CI_Naive_ − CI_1h delay_) / CI_Naive_] was used to normalize for the age-dependent decrease in chemotaxis. ***E***, Short-term aversive olfactory memory assay of wild-type and *msi-1(lf)* mutant worms was tested in young adults and 3-d-old worms as indicated. ***F***, Nose touch response of young, 2-d-old, and 3-d-old wild-type and transgenic animals carrying either SEP::mCherry::GLR-1(wt) or SEP::mCherry::GLR-1(4KR). Data are visualized with Tukey's boxplots. See Extended Data Table 2 for detailed statistical information.

We further investigated whether the unchanged GLR-1 synaptic abundance and dynamics at Day 3 are linked to memory performance in 3-d-old *msi-1(lf)* mutants. To assess memory, we employed a previously described short-term aversive memory (STAM) assay (Vukojevic et al., 2012; Hadziselimovic et al., 2014), which measures attraction toward the chemoattractant DA after aversive olfactory conditioning. We specifically chose STAM over the long-term associative memory assay due to the significant decrease in chemosensation for DA in animals older than 3d, as mentioned earlier (Fig. 1*A*). The STAM assay was performed in 1-d-old and 3-d-old adult animals in wild-type and *msi-1(lf)* mutants. Confirming our previous results (Hadziselimovic et al., 2014), 1-d-old *msi-1(lf)* mutants exhibited better memory performance compared with age-matched wild-type control animals (Fig. 8*E*). Interestingly, 3-d-old *msi-1(lf)* mutants also demonstrated superior memory performance compared with 3-d-old wild-type control animals (Fig. 8*E*).

These results further link the sustained GLR-1 synaptic abundance and turnover dynamics with better memory performance in aged animals. Importantly, *msi-1(lf)* showed a normal life span (Fig. 6*G*), suggesting that *msi-1* specifically affects memory decline rather than influencing aging in general.

### Memory is maintained in GLR-1(4KR) mutants with age

Our experiments strongly suggest that aging alters local GLR-1 dynamics, a process that may be linked to physiological memory decline. Thus, we tested whether the elevation of GLR-1 abundance at synapses during aging can rescue memory performance in aged animals. We performed the STAM assay in young and 3-d-old adult animals in wild-type, GLR-1(WT) overexpressing (SEP::mCherry::GLR-1), and GLR-1(4KR) mutant strain (SEP::mCherry::GLR-1(4KR)). One-day-old SEP::mCherry::GLR-1(WT) and SEP::mCherry::GLR-1(4KR) animals showed similar performance to age-matched wild-type control animals (Fig. 8*A*,*D*). In line with our hypothesis, 3-d-old animals expressing GLR-1(4KR) mutant form exhibited a significant increase in STAM retention compared with their wild-type counterparts [N2 and SEP::mCherry::GLR-1(WT); Fig. 8*B*–*D*]. Calculating the learning and memory indices to compensate for chemotaxis decline revealed that memory, rather than learning, is impaired in 3-d-old animals (Fig. 8*C*,*D*).

Furthermore, we investigated whether this AVA-specific elevated GLR-1 synaptic abundance in the GLR-1(4KR) mutant strain could rescue the gradual age-dependent decline in the nose touch response. We performed the assay in SEP::4KR mutants and compared the performance with aging in SEP::WT animals. To our surprise, we did not observe any significant improvement in SEP::4KR animals on Day 3 compared with SEP::WT animals (Fig. 8*F*).

Altogether, our result suggests that the reduction of the GLR-1 receptor availability at the synaptic membrane during aging is tightly linked to physiological age-dependent memory decline.

## Discussion

In the current study, we have investigated physiological age-dependent changes in GLR-1 abundance, transport, dynamics, and their relation to age-dependent memory decline. We have previously shown that the AMPA-type GLR-1 plays a crucial role in both learning and memory performance in *C. elegans*. Specifically, we demonstrated that GLR-1 in the AVA interneuron pair is essential for memory retention (Vukojevic et al., 2012). AVA interneuron regulates backward movement and also seems to be a critical member of the neural circuit regulating short- and long-term memory formation, as well as maintaining aversive olfactory memory. In addition, *C. elegans* shows age-dependent memory decline, and AVA neuron seems to play an essential role also during this process (Fenyves et al., 2021). Finally, a decline in neuronal plasticity has been proposed to underlie age-dependent cognitive decline (Burke and Barnes, 2006; Bishop et al., 2010). Altogether, these previous results strongly suggest that aging could affect GLR-1 expression, transport, and dynamic properties in general and as a consequence may alter age-dependent cognitive decline through a reduction of responsiveness of the AVA interneuron.

It should be noted that this study focuses on GLR-1-specific age-related changes only in AVA interneuron, while the described changes are likely specific not only to AVA interneuron but also to all GLR-1-expressing neurons in *C. elegans*. On the other hand, the ubiquitination-defective GLR-1(4KR) mutant is driven by the *rig-3* promoter that overlaps in its activity with GLR-1 expression only in AVA neurons. The better memory performance of GLR-1(4KR) mutants observed, compared with the age-matched control, shows that GLR-1 expression and protein stability in AVA interneuron is likely sufficient for memory function at advanced age. These results strengthen our previous findings on the importance of GLR-1 for memory formation in AVA interneurons (Vukojevic et al., 2012).

Using a transgenic line expressing SEP::mCherry::GLR-1, we showed that the abundance of the total AMPA-type glutamate receptor GLR-1 as well as the availability of membrane-bound receptors is significantly reduced with age. As most receptors are produced in the cell soma (Henley and Wilkinson, 2016), one logical explanation would suggest that the reduction of GLR-1 in the AVA axon is caused by reduced GLR-1 protein synthesis or decrease in long-distance transport from the cell body along the axon. However, by measuring the GLR-1 transport frequency, we observed no decline in GLR-1 transport with aging. Furthermore, we also measured the abundance of total GLR-1 in the AVA soma, and, interestingly, we found that GLR-1 is not reduced but, on the contrary, increased in aged animals as compared with young ones. This indicates that the observed decline in GLR-1 abundance in the AVA axon is likely caused neither by a decreased protein production in the cell body nor by reduced transport along the axon to the synapses. The observed reduction in local receptor content in AVA could more likely be caused by a general increase in protein degradation, reduced lateral diffusion, increased local removal, or alternatively decreased local receptor synthesis. The observed increase in receptor synthesis in the soma with age could represent a compensatory mechanism to contrast these processes. In this scenario, unchanged receptor transport could be the limiting factor that explains why the quantity of delivered receptors to the synaptic sites, measured using FRAP by quantifying mCherry signal recovery after photobleaching, is reduced with aging and why GLR-1 could be stalled in the soma. We also detected a significant increase in the ratio of membrane-bound GLR-1 to total GLR-1 with age. This suggests that, as animals age, the mechanism of receptor integration at synapses may not be as strongly affected in 3-d-old animals as the pool of available GLR-1 receptors. This difference could be attributed to a compensatory mechanism or the selective maintenance of essential synapses to ensure a sufficient number of membrane-bound receptors for synaptic transmission.

In addition, we observed that local receptor dynamics change with age. Our results show that with age, the local availability of GLR-1 receptors to the synapse and also the membrane insertion of GLR-1 receptor reduced significantly.

To test whether reduced GLR-1 abundance affects GLR-1 dynamics and memory performance, we used a system in which GLR-1 synaptic abundance is maintained with age. Ubiquitination of GLR-1 receptors has been reported to regulate synaptic strength and the formation or stability of GLR- 1-containing synapses (Grunwald et al., 2004). We found that maintaining the GLR-1 synaptic abundance by using a ubiquitination-defective variant of GLR-1 restores GLR-1 dynamics in 3-d-old animals, indicating that when degradation of GLR-1 is inhibited and receptor abundance does not decrease with age, receptor dynamics also remain intact. Therefore, we hypothesize that a decrease in the axonal total GLR-1 abundance during aging limits the availability of receptors at the synapse, culminating in reduced receptor dynamics and ultimately affecting the plasticity of the system.

We have previously identified several key genes and proteins that influence memory performance in *C. elegans*. For example, the *msi-1* gene encoding an RNA-binding protein from the Musashi family regulates forgetting via translational repression of the Arp2/3 acting-branching complex (Hadziselimovic et al., 2014). *msi-1(lf)* mutants have improved long-term memory retention and, interestingly, do not show age-dependent memory decline (Mastrandreas et al., 2022). It has been shown that inhibition of the Arp2/3 complex leads to a decreased removal of AMPARs from synapses and suppressed LTD (Rocca et al., 2008). These previous findings are in line with our hypothesis, as we observed that GLR-1 abundance and dynamics are unchanged in aged *msi-1(lf)* mutant animals, as opposed to the wild type. Furthermore, in correlation with these results, 3-d-old *msi-1(lf)* mutant animals demonstrated superior memory performance compared with age-matched wild-type control animals. Our experiments also ruled out the possible effect of *msi-1(lf)* mutation on longevity or increased baseline total GLR-1 abundance. Taken together, these findings reinforce our hypothesis that diminished GLR-1 abundance with age not only reduces receptor dynamics but also, crucially influences memory performance in aging animals. Finally, using behavioral assays in a mutant expressing a ubiquitination-defective form of GLR-1(4KR), we could show that blocking GLR-1 degradation improves the memory of aged animals. Altogether, our findings demonstrate that the decrease of GLR-1 levels at synapses during physiological aging is primarily responsible for a gradual decline in memory performance.

On the contrary, AVA-specific elevated GLR-1 synaptic abundance in aged GLR-1(4KR) mutant strain was insufficient to rescue the observed gradual age-dependent decline in the nose touch response. This is likely caused by the fact that the GLR-1-dependent nose touch response is mediated by the ASH, OLQ, and FLP sensory neurons (Kaplan and Horvitz, 1993).

These results support our model that during physiological aging, synaptic GLR-1 abundance is reduced, thus affecting the receptor dynamics and limiting the availability of GLR-1 receptors for membrane insertion. As a consequence, this reduces the efficacy of excitatory neurotransmission by decreasing the number of AMPARs at the surface of synapses (Huganir and Nicoll, 2013; Henley and Wilkinson, 2016). Finally, restoring local GLR-1 levels could serve as an experimental means of preventing age-dependent memory decline.

## Synthesis

Reviewing Editor: Nathalie Ginovart, University of Geneva Decisions are customarily a result of the Reviewing Editor and the peer reviewers coming together and discussing their recommendations until a consensus is reached. When revisions are invited, a fact-based synthesis statement explaining their decision and outlining what is needed to prepare a revision will be listed below. The following reviewer(s) agreed to reveal their identity: Christopher Gabel. Note: If this manuscript was transferred from JNeurosci and a decision was made to accept the manuscript without peer review, a brief statement to this effect will instead be what is listed below.

Synthesis:

Both reviewers expressed great interest in the manuscript and agreed on the significance of the work as a novel contribution to the field. However, the second reviewer raised some concerns that need to be addressed in a revision

Please find the specific comments and queries raised by the reviewers below:

Reviewer 1:

This is a very interesting study documenting how the GLR-1 AMPA type glutamate receptor is affected by age in a key interneuron controlling behavior and memory in C. elegans. It finds a local reduction of GLR-1 in the AVA neuronal processes of older animals and demonstrates that blocking this process helps to increase memory retention in older animals. Thus this study contributes to our understanding of AMPA receptor dynamics and how this relates to decline of memory with age.

This is a much improved revision of the earlier manuscript with errors corrected, additional analysis and explanation. The only remaining factor is the additional statistical information such as "Data S1-H" referred to in the figure legends. It is not clear where this data is (see Statistics section below). Once this is cleared up the study should be suitable for publication.

Statistics section : Figure legends refer to additional statistical information such as "Data S1-H" but it is not obvious where this data is? It does not seem to be in the text or in the statistical table file. I apologize if I missed it but it is not immediately clear where this additional statistical information is.

Reviewer 2:

The goal of this research was to investigate how the dynamics of AMPA-type glutamate receptors change with age and affect memory decline using the nematode C. elegans. To do this they examined the in vivo properties of the AMPA type glutamate receptor subunit GLR-1 in the AVA interneuron using a short-term memory assay pairing diacetyl and starvation and then imaging aspects of glr-1 distribution and function. They found that both total and membrane-bound GLR-1 receptor levels decrease with age in wild-type worms. Using fluorescence recovery after photobleaching (FRAP), they showed that a reduction in GLR-1 abundance correlated with decreased local, synaptic GLR-1 receptor dynamics

This is an interesting study that provides new data about the effects of aging on glr-1 distribution and function. The data is sometimes over interpreted and for a paper with a strong conclusion about a behavior (memory) there are very little behavioral data. There is no clear evidence that this a mechanism of age-related forgetting as they looked at only 2 timepoints and didn't look at multiple ages, or even older worms. The assay used was a short-term memory assay, not a long term memory assay- is the loss one of memory or impaired acquisition (data in fig 8 suggests acquisition is normal, but short-term memory is altered but more attention should be placed on this distinction).

Methods: There needs to be a clearer description of what '3-day-old' vs 'young/1-day-old' worm refers to as it does not outline how they determined that age (from hatch? from egg-laying adult? were there anatomical markers?) and the information in methods is unclear (time from egg lay? incubation temp?).

Please indicate the transgenic strains employed in each of the imaging experiments and indicate whether the behavioral data and the imaging date were collected by an experimenter blind to the strains and/or the hypothesis of the experiment.

Despite the fact that worms are not responding to diacetyl in the later ages (Fig 1A), they seem to be responding enough (positive chemotaxis index) to test if an associative avoidance to DA can be generated with pairing with starvation. What does the CI look like for worms of these ages trained in the starvation paradigm? A graph showing CI for naïve and for trained worms across these ages would be needed to conclude there is an age-related decrease in memory rather than an age related decrease in chemotaxis or locomotor ability.

Are there any old-age associative chemotaxis studies- if so, they should be reviewed here? To confirm age-related decline in memory the authors should have looked in older worms and compared the performance of untrained vs trained worms.

Related to the what is the rationale for using the diacetyl assay? Associative aversion to NaCl provides a wider range of attraction/avoidance behavior (as this author has previously reported Stetak et al., 2009) and could offer the ability to look at learning at later life stages of the worm, especially since they report in the supplemental material that worms survive at least 8 - 10 days of adulthood before they start to die off.

What is the E. coli consumption rate older worms, if it decreases with age, starvation may not be able to drive aversion as much in older worms?

There are clearly age-related changes in glr-1 abundance and turnover at 3 day old adults- to conclude that this is a learning/memory specific change they would need to investigate other glr-1 dependent behaviors to see if they are also altered.

On pg 17 the authors state "These results with msi-1 further link the sustained GLR-1 synaptic abundance and turnover dynamics with better memory performance in aged animals.... suggesting that msi-1 specifically affects memory decline rather than influencing ageing in general." In the abstract it says "Genetic manipulation of GLR-1 stability, either by deleting msi-1 or expressing a ubiquitination-defective GLR-1 (4KR) variant, prevented this age-related reduction in receptor abundance and improved memory performance in older animals, which reached performance levels similar to those of young animals." They showed the memory effect for the GLR-1(4KR) but did not show is for the msi-1. A figure like figure 8 showing that msi-1 worms do not show the memory deficit would allow this conclusion.

Finally, the title is misleading as they are training and testing the animals at the same age so the longest retention being tested is 1 hour. 'Age-related' memory decline suggests decay of lasting memory. This may be a defect in 'learning' or 'short-term memory' rather than simply memory in the title. A more appropriate title would emphasize the age-related changes in glr-1, and that these changes correlate with (rather than drives- it is only a correlation) alterations in memory.
